# National research for health systems in Latin America and the Caribbean: moving towards the right direction?

**DOI:** 10.1186/1478-4505-12-13

**Published:** 2014-03-06

**Authors:** Francisco Becerra-Posada, Miryam Minayo, Cristiane Quental, Sylvia de Haan

**Affiliations:** 1 Council on Health Research for Development (COHRED), Camino Real a Xochimilco 17-22, Santa Maria Tepepan, México, D.F. 16020, México; 2 CRIS/FIOCRUZ, Av. Brasil, 4365 - Manguinhos, Rio de Janeiro, RJ CEP: 21040-360, Brasil; 3 Escola Nacional de Saúde Pública Sérgio Arouca, Fundação Oswaldo Cruz - FIOCRUZ, Rua Leopoldo Bulhões 1480, CEP 21041-210 Rio de Janeiro, Brasil; 4 Council on Health Research for Development (COHRED), 1-5 Route des Morillons, 1211 Geneva 2, P.O. Box 2100, Switzerland

**Keywords:** Health research, Latin America and Caribbean, National research for health systems

## Abstract

**Background:**

National Research for Health Systems (NRfHS) in Latin America and the Caribbean (LAC) have shown growth and consolidation in the last few years. A structured, organized system will facilitate the development and implementation of strategies for research for health to grow and contribute towards people’s health and equity.

**Methods:**

We conducted a survey with the health managers from LAC countries that form part of the Ibero-American Ministerial Network for Health Education and Research.

**Results:**

From 13 of 18 questionnaires delivered, we obtained information on the NRfHS governance and management structures, the legal and political framework, the research priorities, existing financing schemes, and the main institutional actors. Data on investment in science and technology, scientific production, and on the socio-economic reality of countries were obtained through desk review focused on regional/global data sources to increase comparability.

**Conclusions:**

By comparing the data gathered with a review carried out in 2008, we were able to document the advances in research for health system development in the region, mostly in setting governance, coordination, policies, and regulations, key for better functionality of research for health systems. However, in spite of these advances, growth and consolidation of research for health systems in the region is still uneven.

## Background

Health and health equity are essential conditions for the development of nations, societies, and individuals [1]. The use of research, research findings, research based policies, and innovation are considered key to achieve health, development, and economic growth [2-4]. Increasingly, low- and middle-income countries are beginning to invest in research and innovation [5] – viewing this as a realistic way towards sustainable and country-driven development. Health progress is increasingly tackled as a cross-sectoral issue [6,7], and countries act by increasing their research and innovation budgets, putting in place enabling policies, or by engaging in large collaborative partnerships in research and innovation.

This article reflects on the current status of National Research Systems for Health (NRfHS) in Latin American and Caribbean (LAC) countries. Health research systems (HRS) have been defined as “*the people, institutions, and activities whose primary purpose in relation to research is to generate high-quality knowledge that can be used to promote, restore, and/or maintain the health status of populations*” [8]. Separating the biomedical connotation of the term ‘health research’ has made research in the health field more inclusive; in 2008, during the Global Ministerial Forum on Research for Health [9], the specific nomination of ‘research for health’ was introduced with the intention to “…*link discussions on health research more closely with ongoing developments in science and technology across multiple sectors, including social determinants of health, such as food security, the environment, housing, education, work conditions, income distribution, and social safety-nets*” [6].

NRfHS can provide a systemic approach to decisions to guide and develop health research [8]. They ensure that research agendas are set, policy frameworks are defined, and a structure or mechanism is created that manages the policies, negotiates and contracts with partners, and facilitates the review of national research priorities as part of a coordinated approach. Experiences from countries illustrate the usefulness of addressing research for health from a systems perspective [10-12].

The interest and need to strengthen research for health systems can also be seen in the LAC regions. The first regional approach to understand the development of research for health systems was developed during the 1^st^ Latin American Conference on Research and Innovation for Health [13] that took place in Rio de Janeiro in April 2008. The report highlighted that most of the NRfHS in the region were inefficient, lacked coordination, worked with undefined priorities, had no sustainable financing mechanisms, and the key elements of the system were dispersed and uncoordinated. An analysis of 14 Latin American countries that participated at the conference showed the multiplicity and weak governance structures, coordinating mechanisms, and non-sustainable financing schemes for research for health across the region [10].

Regional- and country-based developments to strengthen research for health continued since then have taken place and include the Regional Policy for Research for Health by PAHO/WHO [14], the work of the Ibero-American Ministerial Network for Health Education and Research (RIMAIS), the development of the Research for Health Commission of the Council of Ministers of Health of Central America and Dominican Republic (COMISCA), and the realization of the 2^nd^ Latin American Conference on Research and Innovation for Health, in 2011, during which new recommendations were issued by ministers, researchers, academicians, and managers of research for health from different ministries and agencies in the region on how to improve research and innovation. These recommendations can be summarized in the following actions: promoting tax exemptions for innovation producers and financers; establishing technology parks; promoting partnerships between academia, government, and manufacturers; using royalties (national funds) to finance research and innovation; facilitating research internships at productive companies; promoting the creation of innovation offices within universities; giving scholarship incentives for innovation exchange; differentiating regulatory stimuli for research and innovation and promoting the implementation of innovation agencies in universities [15].

The regional support to countries to strengthen their NRfHS is a strategy in which common goals can be achieved through collaborative efforts, as well as facilitating exchange of experiences and learning between countries. Having a regional perspective also allows for benchmarking on the advances and allows countries to set plans in order to advance.

In this paper, four years after the first cross-regional analysis [10], and using the same assessment tool, we again review and analyze the research for health systems in the region. We specifically address the progress that has been made during these years, and what is still needed to further strengthen research for health in the region.

## Methods

The situation analysis consisted of two phases: a self-administered questionnaire and a desk review. The questionnaires collected information on national research for health governance and management structures, legal and policy frameworks, research agendas, financing schemes, and the main institutional actors in research for health.

Eighteen questionnaires were distributed to RIMAIS country representatives and 13 were returned by February 2012, providing information on Argentina, Brazil, Costa Rica, Dominican Republic, Ecuador, Guatemala, Honduras, Mexico, Panama, Paraguay, Peru, Uruguay, and the Caribbean as a region. For the Caribbean, the questionnaire was answered by the Caribbean Health Research Council, the sub-regional coordinating body. The questionnaires were distributed at a side meeting of RIMAIS in Panama, during the 2^nd^ Latin American Conference on Research and Innovation for Health held in November 2011, which provided the opportunity to review the instrument and for the lead researchers to respond to questions for clarification. The people that received the questionnaire were typically the ones to complete or lead an internal process to answer the questions on behalf of their respective Ministries of Health (MoHs).

The questionnaire was adapted from the research system development framework of COHRED [16]. The same questionnaire was used in 2008 to collect information from the country participants in preparation for the 1^st^ Latin American Conference on Research and Innovation for Health (Rio de Janeiro, 2008). The analysis of the 2008 survey, comprising information from 14 countries, was published in 2009 [10].

The desk review focused on collecting data on investments in science and technology, scientific production, and data on the socio-economic status of the countries. The following publicly available sources were used: the Economic Commission for Latin America (CEPAL) [17], the United Nations Development Programme [18], Ibero-American Network of Science and Technology Indicators (RICYT) [19], the World Bank [20], the World Intellectual Property Organization [21], SCImago Journal and Country Rank [22], and the Latin American Literature in Health Sciences data base [23].

## Results

### Context: socioeconomic review of LAC countries

To understand the development of health and research for health systems in the region there is a need to understand the socioeconomic situation of the countries. The Latin American region is composed of Spanish-, Portuguese-, Dutch-, French-, and English-speaking countries of the Americas; the Caribbean comprises countries with different languages, and most of them compose the English-speaking Caribbean.

LAC is going through a demographic transition due to low fertility rates and a decrease in mortality rates. Life expectancy has increased by seven years over the last 25 years and now exceeds 70 years [24].

Table 1 lists key indicators for the countries of the region. The total population of the region is over 600 million, 575 million living in Latin America. Average life expectancy is 74.7 years, while country life expectancy ranges between 62.5 for Haiti and 79.6 for the United States Virgin Islands [17].

**Table 1:** Latin American and Caribbean key indicators

**Countries and regions**	**Population (×1,000 at mid-year), 2010**	**Life expectancy (both sexes), 2010–2015**	**Annual growth rate (GDP), 2010**	**Public investment in health (% of GDP current prices), 2009**	**Infant mortality rate (per 1,000 live births), 2010–2015**	**Maternal mortality rate (per 100,000 live births), 2008**
Anguilla	15	…	…	…	…	…
Antigua and Barbuda	89	…	–7.9	…	…	…
Netherlands Antilles	201	76.8	…	…	11.7	…
Argentina	40,738	76.2	9.2	6.2	12.0	70
Aruba	107	75.5	…	9.6	14.0	
Bahamas	343	75.9	0.9	2.3	7.9	49
Barbados	273	77.1	0.2	2.9	9.4	64
Belize	312	76.3	2.9	…	15.2	94
Bolivia	10,031	67.2	4.1	1.9	38.1	180
Brazil	195,498	73.5	7.5	…	20.3	58
Chile	17,133	79.1	5.2	4.1	6.5	26
Colombia	46,299	73.9	4.3	2.2	16.5	85
Costa Rica	4639	79.4	4.2	6.6	9.3	44
Cuba	11,203	79.1	2.1	10.6	4.5	53
Dominica	68	…	0.9	…	…	…
Ecuador	13,773	75.8	3.6	…	17.6	140
El Salvador	6,192	72.1	1.4	4.1	17.5	11
Grenada	104	76.2	0.0	2.8	11.7	…
Guatemala	14,376	71.4	2.8	1.4	22.6	110
Guyana	754	70.3	4.4	9.9	37.0	270
Haiti	10,089	62.5	–5.1	…	43.6	300
Honduras	7,621	73.1	2.8	…	24.9	110
Cayman Islands	56	…	…	…	…	…
Turks and Caicos Islands	38	…	…	…	…	…
British Virgin Islands	23	…	v	…	…	…
United States Virgin Islands	109	79.6		…	8.4	…
Jamaica	2,741	73.5	–1.3	…	21.5	89
Mexico	110,675	77.2	5.6	3.1	13.7	85
Montserrat	6	…	…	…	…	…
Nicaragua	5,822	74.5	4.5	…	18.1	100
Panama	3,508	76.3	7.6	2.2	15.7	71
Paraguay	6,460	72.8	15.0	3.4	28.8	95
Peru	29,495	74.1	8.8	1.1	18.8	98
Puerto Rico	3,749	79.3	…	1.6	6.6	18
Dominican Republic	9,899	73.2	7.8	2.4	25.1	100
St. Kitts and Nevis	52	…	–2.4	4.0	…	…
St. Vincent and the Grenadines	109	72.6	–2.8	…	20.3	…
Saint Lucia	174	74.9	3.2	…	11.2	…
Suriname	525	70.9	4.5	2.5	20.5	100
Trinidad and Tobago	1,341	70.4	0.0	…	23.8	55
Uruguay	3,372	77.1	8.5	5.1	11.5	27
Venezuela	29,043	74.7	- 1.5	…	15.3	68
**Latin America and the Caribbean**	**590,082**	**74.7**	**5.9**		**18.9**	**85**
**Latin America**	**575,867**	**74.6**	**6.0**		**18.6**	
**The Caribbean**	**41,646**	**72.7**	**0.2**		**32.6**	

Source: CEPAL 2011 Anuario estadístico de América Latina y el Caribe [17].

The continent is also undergoing an epidemiological transition. Without underestimating the burden of disease attributable to communicable diseases, at present, non-communicable diseases account for the largest proportion of the burden of disease. These include chronic degenerative diseases, mental disorders, as well as morbidity and mortality resulting from accidents, injuries, and violence [17].

Table 2 shows a series of context indicators that demonstrate the differences between countries in the region. It lists development indicators, investment in research and development and scientific production. The human development index (HDI; prepared by the United Nations Development Program) shows that Chile was the country that had the highest index of the region (0.798) in 2009. In that year, the average of the region’s HDI was 0.722. Argentina, Uruguay, Cuba, Mexico, Panama, and Costa Rica all had an HDI above the regional average [18] (Table 2).

**Table 2:** Relevant aspects regarding development, investment and scientific research in evaluated countries

**Country**	**Context indicators**			**National commitment to health and education**		**General countries data expenditures and production of technology and knowledge**					**Publications of scientific research**			
	**Human development**^**a **^**(IDH)**	**Population (millions)**^**b**^	**GDP (current in billions of US$ )**^**c**^	**Public expenditure on health (%GDP)**^**d**^	**Public expenditure on education (%GDP)**^**e**^	**Expenditures on science and technology activities (per capita) US$**^**f**^	**Expenditures on experimental R&D (per capita) US$**^**g**^	**Patents granted to residents (by millions of individuals)**^**h**^	**Royalties and license fees, receipt (by millions of individuals) US$**^**i**^	**Researchers in R&D (by millions of individuals)**^**j**^	**Scientific publications indexed by LILACS**^**k**^	**Scientific publications indexed by MEDLINE (% of all publications indexed in MEDLINE)**^**l**^	**Publications in all fields of science indexed by the SCI (No.)**^**m**^	**Scientific publications in the health field indexed by SCI (No) (% of publications in all fields)**^**n**^
	**2009**	**2009**	**2009**	**2009**	**2009 or last available year**	**2009**	**2009**	**2009 or last available year**	**2009**	**2007**	**2009**	**2009**	**2008**	**2008**
Argentina	0.788	40.1	307.1	5.1	6	51.5	46.05	6.14 (2008)	2.7	980	1,051	2,456 (0.32)	6,197	3,531 (56.98)
Brazil	0.708	193.3	1,594.5	3.5	5.4 (2008)	130.53	88.84	1.76	2.2	657	15,945	13,335 (1.74)	26,482	17,792 (67.19)
Costa Rica	0.738	4.6	29.3	5.9	6.3	147.13	35.35	…	0.1	…	76	99 (>0)	..	…
Dominican Rep	0.68	9.8	46.8	1.9	2.3	…	…	…	…	…	…	2 (>0)	…	…
Ecuador	0.716	14,261.6	109.16	2.3	4.9	209.6	140.69	0	…	106	35	48	…	…
Guatemala	0,569	14	37.7	2.1	3.2 (2008)	…	1.49	0.07	0.9	29	14	27 (>0)	…	…
Honduras	0.619	7,449.9	27.65	4.1	0	…	…	1	…	…	35	11	…	…
Mexico	0.762	112	879.7	2.7	4.9 (2008)	31.61	32.39	1.9	…	353	508	2,949 (0.38)	8,262	4,329 (52.40)
Panama	0.76	3.5	27.7	4.3	3.8 (2008)	35.38	14.52		…	144	9	41 (>0)	…	…
Paraguay	0.651	6.3	14.2	2.4	4.0 (2008)	…	…	3.58 (2007)	41.9	..	35	10 (>0)	…	…
Peru	0.714	28.8	126.9	2.5	2.6	…	…	…	0.8	…	35	10 (>0)	…	…
Uruguay	0.773	3.4	31.3	5.9	2.8 (2006)	61.68	40.12	0.45		…	189	194 (>0)	…	…
LAC	0.772					76.1	44.08	0.88	0.0^**^	443 (^***^)	22,035	21,954 (2.88)	48,791	30,478 (62.47)

…Data not available.

**Greater than zero, but not enough to be rounded to zero.

***Source: [25].

^a^and ^b^Source: [18]; ^c^The numbers were rounded. Source: [19]; ^d^Source: [18]; ^e^Source: [25]; ^f^and ^g^Source: [19]; ^h^Source: [21]; ^i^and ^j^Source: [20]; ^k^and ^l^Source: [19]; ^m^and ^n^Source: [25].

Five countries were responsible for 80% of the GDP of the region in 2009 – Brazil, Mexico, Argentina, Venezuela, and Colombia (Table 3) [19]. This concentration draws attention to the need for different development strategies, which in turn will impact the type of policies and activities on science, technology, and innovation adopted for each country in the region.

**Table 3:** Distribution of GDP (in PPP in US$ billion) in Latin America, 2009

**Countries**	**Distribution of GDP (in PPP in US$ billion)**	**13 Countries accumulate 95% of the GDP**
Brazil	2,040	33.6
Mexico	1,466	57.8
Argentina	585	67.5
Colombia	410	74.3
Venezuela	337	**79.9**
Peru	252	84
Chile	240	88
Ecuador	111	89.8
Puerto Rico	96	91.4
Dominican Rep.	80	92.7
Guatemala	70	93.9
Costa Rica	49	94.7
Bolivia	46	**95.5**
Uruguay	44	
El Salvador	42	
Panama	40	
Honduras	32	
Paraguay	28	
Trinidad and Tobago	27	
Jamaica	24	
Nicaragua	17	
Haiti	12	
Barbados	5	
Guyana	5	
**TOTAL LAC**	**6,058**	

Source: RICYT, 2012. Indicators. Gross Domestic Product [18]. PPP = Parity Purchase Power.

### Assessment of the NRFHS in LAC countries

#### ***Governance and management***

Seven out of the 12 respondent countries in Latin America as well as the Caribbean as a region stated having a formal governance body dedicated to research for health. The Health and Science and Technology Ministries/Secretaries usually share this governance with either specific responsibilities (mainly financing), in a structured system, or lead the scientific sector when there is not a system in place.

The Health sector, through the MoHs, is often responsible for governing (setting policies, priorities, financing, etc.) clinical and public health research and uses the country’s health policy or plan to set directions for it (Table 4). The Science and Technology (S&T) sector represented by specialized agencies which have a different status in each country, such as at MoH level in Brazil and Argentina, or Council in the rest, is mostly responsible for basic and biomedical research financing, as well as efforts towards innovation. It is common for both sectors to have their own management structures and priorities for research. Coordination between the two areas — which is crucial for an NRfHS — varied between the respondent countries. Respondents indicated that for the most part, coordination between the two sectors takes place through the participation of a representative of the Health sector in the sectoral group “health” within the S&T sector. However, given that MoHs or Councils are the ones with most funding available, they play a key role in deciding the topics to finance. The level of coordination between MoHs and S&T will have a direct impact in co-financing a mutually agreed research agenda and, thus, advance the research for health actions in any given country.

**Table 4:** Formal bases of the NRfHS in analyzed countries

**Country**	**Governance**	**Specific policy**	**Laws and regulations**	**Priorities**
Argentina	Yes	No	Yes	Yes
Brazil	Yes	Yes	Yes	Yes
Costa Rica	Yes	Under development	Yes	Yes
Dominican Republic	Yes	Pending approval	Pending approval	No
Ecuador	Yes	Yes	Under development	Yes
Guatemala	Yes	No	Yes	Yes
Honduras	No	No	No	No
Mexico	Yes	Governmental agreement	Yes	No
Panama	No	No	Yes	No
Paraguay	Under implementation	Approved, under implementation	Under implementation	Yes
Peru	Yes	No	Yes	Yes
Uruguay	No	No	No	No
Caribbean Health Research Council	Yes	Yes	No	Yes

Source: Applied surveys.

Regarding polices, a cornerstone for structuring a NRfHS, some countries in the region, such as Brazil, Ecuador, and Paraguay, have developed specific policies for research for health, where in Paraguay the policy was issued by Presidential decree [26] and is in the process of implementation. Other countries are discussing and approving such policies; the Dominican Republic has a draft policy and expects it to be approved in the near future and in Costa Rica it is under development. Peru has issued a series of guidelines and manuals for better operation of research for the health environment [27]. Argentina, Guatemala, Honduras, Mexico, Panama, and Uruguay have no specific policies, but general guidance and objectives for research for health are provided through national programs and could be understood as a policy. Nonetheless, most countries that completed the questionnaire indicated having laws and regulations for clinical studies, ethical standards for research, and product registries.

In the Caribbean, research for health is under the mandate of the Caribbean Health Research Council (CHRC), through the newly created Caribbean Public Health Agency (CARPHA), that supports and coordinates research in all English-speaking Caribbean countries and has a sub-regional policy for research for health [28].

### Priorities in research for health

Among the completed questionnaires, eight reported having a specific agenda of national priorities in research for health – Argentina, Brazil, Costa Rica, Ecuador, Guatemala, Paraguay, Peru, and the Caribbean region (Table 5). The processes were all participatory and used different approaches on how to involve stakeholders, from local to national meetings in Brazil, to Internet-based participation in the Caribbean, and workshops and meetings in other countries.

**Table 5:** Countries with national priorities

**Country**	**National priorities**	**Year**	**Lead institution**
Argentina	Yes	2012	Ministry of Health and Ministry of Science and Technology
Brazil	Yes	2011	Ministry of Health
Costa Rica	Yes	NA	Ministry of Health
Dominican Republic	No		
Ecuador	Yes	NA	Ministry of Health
Guatemala	Yes	2013	Ministry of Health
Honduras	No		
Mexico	No		
Panama	No		
Paraguay	Yes	2008	Ministry of Health
Peru	Yes	2009	National Institute of Health
Uruguay	No		
Caribbean Region	Yes	2010	Caribbean Health Research Council

Leading institutions were mainly MoHs. In Peru, it was lead by the National Institute of Health, which reports to the MoH. In Argentina, both the MoH and the Ministry of Science and Technology, have developed priorities for research for health. In the Caribbean, it was coordinated by the CHRC [29-35].

Five countries, the Dominican Republic, Honduras, Panama, Mexico, and Uruguay indicated that they have not established any national priorities for research for health (Table 5). However, Mexico has a general listing of topics from where research topics are selected in a collegiate manner for the issuing of calls for proposals by the Council of Science and Technology.

### LAC investment and financing in research for health

There are many difficulties in measuring and comparing research and development (R&D) investments in the region. Available data is not up to date and there are concerns with regard to methodology and data accuracy. Data shows overall expenditures in R&D and is not disaggregated for the different areas being financed. According to data obtained through the desk review, it is estimated that the investments in R&D in the LAC region rose from US$14.4 billion in 2005 to US$26.9 billion in 2009. In 2005, Brazil accounted for over half of the investment in R&D in the region [19], and together with the investments of Mexico and Argentina, the three countries represent 89.7% of the R&D investment of the whole region. In 2009, Brazil was the country that invested the most in R&D as a percentage of its GDP, with 1.18%, while the average in the region was 0.69% (Table 6). From 2005 to 2009, Brazil increased its investments reaching 69% of the total of the LAC region, while Mexico reduced its national R&D investment by half (Table 7) [22].

**Table 6:** Investments in R&D with regard to GDP (%)

**Regions and countries**	**Investments in R&D with regard to GDP (%)**
USA	3.04%
Canada	1.92%
Spain	1.38%
Brazil	1.18%
Ibero-American	0.88%
LAC countries	0.69%
Cuba	0.64%
Argentina	0.59%
Costa Rica	0.54%
Uruguay	0.42%
Mexico	0.39%

Selected regions and countries, 2009.

Source: RICYT, 2012. Indicators, Expenditure on R&D [18].

**Table 7:** Distribution of the investment in R&D in billions of US$ in the LAC countries

**Country/Year**	**2005**	**%**	**2009**	**%**
Argentina	844	6	1846	7
Brazil	8564	60	18918	69
Mexico	3496	24	3887	14
Total LAC	14342	100	27336	100

Source: RICYT, 2012. Indicators. Expenditure on Science and Technology [18].

Few countries reported having information on the financing data for research for health. Results from the questionnaires indicate that country level investments in research for health mostly come from public funds. The main financers are the Ministries or Councils of S&T through their various programs. Health financing mechanisms as well as financing agencies are growing in importance, especially in Brazil. Some countries have established sustainable mechanisms to secure financing for research and to break the dependency on annual budgetary cycles and variations. Argentina, Brazil, Chile, Mexico, and Uruguay finance research through sectoral funds, while Paraguay is considering such a financing mechanism [36,37].

In the other countries though, there is almost no local financing for research with research funding overwhelmingly obtained from external sources and grants.

The interest from the WHO and its Member States on increasing the financing for research for health and to implement a tracking system of financial flows has been recently expressed by the Executive Board of the WHO, and is expected to be followed up in coming sessions and special meetings [38]. The financing issue was specifically expressed during the Mexico Ministerial Summit on Health Research (Mexico City, 2004), followed by a resolution from the World Health Assembly [39,40]. So far, few countries have been able to fulfil this goal.

## Discussion

The development and consolidation of NRfHS in the region has been gaining momentum, mainly in the second half of the last decade. At a regional level, a main breakthrough was the approval by all member states in 2009 of the Regional Policy for Research for Health by PAHO/WHO. It calls for national strengthening and/or development of NRfHS with all the needed foundations for its growth and optimization. The opportunity to discuss advances in the development of their countries, to compare these with the advances in other countries, and to issue recommendations through the reports of the first and second Latin American Conferences on Research and Innovation for Health have favored exchange and knowledge sharing.

As a result of the actions described and the vision of governments in the region during the last decade, most of the countries that responded to the survey reported making important investments and advances in their NRfHS. This progress can be attested by comparing the 2012 survey results with those of 2008. These investments are reflected in the strengthening and development of their respective NRfHS, with noticeable results that are mostly reflected in governance, coordination, and policies.

Eight of the 13 questionnaires received showed countries with a formal governance body dedicated to research for health, specific policies and/or a research for health program, and a plan of priorities for research in health defined by participatory processes; many other countries are on the way. The developments over these past four years show that most countries have set in place laws/regulations for clinical studies, and ethical standards for research and product registration.

One of the countries with the most advances as a result of having a new policy is Paraguay, where they have set in place the National Researchers’ System. This system aims to stimulate researchers’ productivity through curricular evaluation and the allocation of economical stipends for those approved. This same scheme has been operating in Mexico for a long time and has proven to be an efficient way to stimulate researchers and to compensate the low salaries many these have.

Financing mechanisms for research for health are being enhanced, mainly in Brazil, Mexico, Argentina, and Chile. However, the growth for financing has been slow in other LAC countries. This is an area of opportunity for countries to share experiences and further advance in developing sustainable financing mechanisms. There is an important lack of financing for research for health in the countries in the region, probably due to the fact that governments still do not understand the value of research and the relevance for the health of their populations.

Some of the key issues when using public funding are transparency and accountability. Peru and Argentina have strengthened their systems and are implementing strategies for better coordination and accountability of research funded with public funds. The centralized call for proposals in many countries and the different mechanisms that have been established to secure the transparency in the allocation process contribute to this goal and offer researchers and institutions a co-responsibility in the use and reporting of funds and research results.

In the Caribbean, the role of the CHRC has been crucial to maintain research for health in the region. Having simultaneous policies and a prioritized research agendas is only the first step towards developing (or creating) stronger systems. Even though the CHRC is the oldest research body in the Americas, it does not have a wide funding mechanism to secure financing to implement the research agenda and will have to develop other strategies to ensure sufficient allocation of funds to priority areas for the region, which is now part of the recently created CARPHA.

Sub-regional efforts play a key role in moving forward towards strengthening research for health in one of the most needed areas in the region. The Executive Secretariat for the COMISCA has installed the Technical Commission for Research for Health to support the development and implementation of national and regional activities towards strengthening NRfHS in the sub-region.

The countries in the region need to implement further changes in their NRfHS in order to advance in their social, equity, and heath improvement. There are countries that have advanced in implementing coordinating structures and mechanisms that required more political will and vision than funding. It is urgent to engage other countries in the improvement of their systems as to increase their growth in research and innovation for health. Research should also be seen as an innovation component towards economic growth.

All countries have some parts of the system in place, but these are uncoordinated and disaggregated. Consequently, a focus should be placed on developing or strengthening the foundations of a system to operate (policies, priorities, and management), so as to be able to move towards capacity building/strengthening, and establishing financing and coordinating mechanisms. This would help to enable systems to move towards optimization in order to achieve their full potential.

As stated, there is an opportunity for countries in the region to learn from each other from the different successes they have achieved. Some of these exchanges have already happened and successful initiatives have been set in place in Paraguay.

## Conclusions

There have been advances over a 4-year period between reports on the NRfHS status. The interest that the Latin American Conferences on Research and Innovation for Health have raised, the Policy on Research for Health by PAHO, the work of RIMAIS and recently of COMISCA, and the continuous support from COHRED, have contributed to these developments.

Growth and consolidation of NRfHS in the region is uneven, however. There is much work ahead and many needs. Adopting a system’s approach to support NRfHS strengthening, rather than promoting individual or isolated capacity building actions, would render better and sustainable benefits to the people of the region.

It is crucial that countries in the region keep up the momentum and invest in strengthening their research and innovation systems for health. If the full potential of research for health is to be achieved, there is an urgent need to develop, strengthen and consolidate NRfHS for its optimal operations. A NRfHS needs to have, at a minimum, a defined policy or program, linked to national priorities for research for health, a coordinating and managerial structure, a sustainable financing mechanism, and defined monitoring and evaluation indicators. A NRfHS is the sum of efforts and responsibilities of different actors in each country, each doing and exercising their own mandates but in a coordinated way so as to benefit from the full potential of research infrastructure and of research results that can be transferred into policies and programs.

## Abbreviations

CARPHA: Caribbean Public Health Agency; CHRC: Caribbean Health Research Council; COMISCA: Council of Ministers of Health of Central America and Dominican Republic; HDI: Human development index; HRS: Health research systems; LAC: Latin American and Caribbean; NRfHS: National Research Systems for Health; MoH: Ministries of Health; RIMAIS: Ibero-American Ministerial Network for Health Education and Research; R&D: Research and development; S&T: Science and technology.

## Competing interests

The authors declare that they have no competing interests.

## Authors’ contributions

Questionnaire design (SdH, FBP), Analysis (MM, CQ), Manuscript draft (FBP, MM, CQ), Manuscript reviews (FBP, MM, CQ, SdH). All authors read and approved the final manuscript.

## Collaborative group

Jackeline Alger, María Alejandra Alvarez, Melany Ascencio, Eduardo Artz, Cleidemar Batista Valério, Greice Stefani Borghetti, Patricia Caballero, Jailson de Barros Correia, Marcia Luz da Motta, Malvina Hortensia de León, Emilia Guzmán, Ignacio Ibáñez, Ramiro Iván Lopez Pulles, Lizbeth Martínez de Benítez, Carolina O’Donnell, Ana Perez, Sonia Patricia Romano Riquer. Luis Santa Maria, Mónica Silenzi, Donald Simeon, and Luis Tacsan.
